# Taurine Alleviates Chronic Social Defeat Stress-Induced Depression by Protecting Cortical Neurons from Dendritic Spine Loss

**DOI:** 10.1007/s10571-022-01218-3

**Published:** 2022-04-18

**Authors:** Yuanyuan Zhu, Rui Wang, Ze Fan, Danlei Luo, Guohong Cai, Xinyang Li, Jiao Han, Lixia Zhuo, Li Zhang, Haifeng Zhang, Yan Li, Shengxi Wu

**Affiliations:** 1 Department of Neurobiology, The School of Basic Medicine, The Fourth Military Medical University, Xi’an, 710032 Shaanxi China; 2 Department of Anesthesiology & Center for Brain Science, The First Affiliated Hospital of Xi’an Jiaotong University, Xi’an, 710061 Shaanxi China; 3 State Key Laboratory of Military Stomatology, Department of Anesthesiology, School of Stomatology, The Fourth Military Medical University, Xi’an, 710032 Shaanxi China

**Keywords:** Taurine, Depression, mPFC, Dendritic spine, NMDAR

## Abstract

Abnormal amino acid metabolism in neural cells is involved in the occurrence and development of major depressive disorder. Taurine is an important amino acid required for brain development. Here, microdialysis combined with metabonomic analysis revealed that the level of taurine in the extracellular fluid of the cerebral medial prefrontal cortex (mPFC) was significantly reduced in mice with chronic social defeat stress (CSDS)-induced depression. Therefore, taurine supplementation may be usable an intervention for depression. We found that taurine supplementation effectively rescued immobility time during a tail suspension assay and improved social avoidance behaviors in CSDS mice. Moreover, taurine treatment protected CSDS mice from impairments in dendritic complexity, spine density, and the proportions of different types of spines. The expression of *N*-methyl d-aspartate receptor subunit 2A, an important synaptic receptor, was largely restored in the mPFC of these mice after taurine supplementation. These results demonstrated that taurine exerted an antidepressive effect by protecting cortical neurons from dendritic spine loss and synaptic protein deficits.

**Supplementary Information:**

The online version contains supplementary material available at 10.1007/s10571-022-01218-3.

## Introduction

With societal and economic advances, people are more likely to suffer from psychiatric and dysthymic disorders characterized by a range of symptoms, such as depressed mood, anhedonia, social avoidance, cognitive impairment, and sleep disturbances (Banasr et al. 2021; Atique-Ur-Rehman and Neill 2019; Liu and Thompson 2017). However, the molecular pathogenesis of depression is not fully clear. Typical antidepressant medications, such as tricyclic antidepressants, monoamine oxidase inhibitors, and selective serotonin reuptake inhibitors, may not show the desired effects for at least three weeks, and many patients do not respond to these treatments (Kupfer et al. 2016; Shulman et al. 2013; Feighner 1999). Therefore, the identification of novel mechanisms and targets is urgently needed to design corresponding therapeutic strategies for major depressive disorder (MDD).

Taurine, a sulfur-containing amino acid, is known to be semiessential for mammals. In recent decades, taurine has been reported to have many physiological and pharmacological functions, including maintaining membrane stabilization, attenuating inflammation- and oxidative stress-induced injuries, modulating endoplasmic reticulum stress and osmotic pressure, maintaining calcium homeostasis, and acting as a trophic factor in the central nervous system (Menzie et al. 2014). Taurine can be transported into the brain through the taurine transporter (TAUT), which is widely expressed in the brain parenchyma and blood–brain barrier (BBB). The level of taurine has been reported to be decreased in the brains of depressive rats, suggesting that taurine is involved in the development of depression (Wu et al. 2017). However, whether and how taurine supplementation is beneficial for MDD patients remains largely unknown.

In this study, we found significantly reduced levels of interstitial taurine in the cerebral medial prefrontal cortices (mPFC) of chronic social defeat stress (CSDS) mice (Golden et al. 2011). In addition, depression-like behaviors, dendrite morphology, and synapse-associated protein expression were further evaluated in CSDS mice after taurine treatment.

## Materials and Methods

### Animals

Male C57BL/6 J mice (∼ 23 g) and male CD-1 mice aged 7–8 months (∼ 45 g) were purchased from Beijing Weitong Lihua Experimental Animal Technology Co., Ltd. (China). All mice were housed at a temperature of 22 ± 2 °C on a 12 h light and 12 h dark cycle with food and water available ad libitum. All animal handling and experimental procedures were performed in accordance with the Animal Welfare Act and the Guide for the Care and Use of Laboratory Animals and were approved by the Animal Care and Use Committee of the Fourth Military Medical University (ID: 20171202).

### Experimental Procedures

Experiment 1: C57BL/6 J mice were separated into two groups: a control group (*n* = 9) and a CSDS group (*n* = 9). Experiment 2: C57BL/6 J mice were separated into three groups for CSDS and taurine treatment: the control group (*n* = 8–9), saline-treated CSDS group (*n* = 5–7), and taurine-treated CSDS group (*n* = 8–10). C57BL/6 J mice were intraperitoneally (i.p.) injected with taurine (500 mg/kg) or an equal volume of 0.9% saline once a day for 10 consecutive days. The taurine dose was based on previous reports (Wu et al. 2017; Samadi et al. 2021; Heidari et al. 2019). We then conducted behavioral experiments and collected brain samples, which were frozen in liquid nitrogen and stored at − 80 °C. The taurine content of brain samples was detected using a Taurine Assay Kit (Cell Biolabs, MET-5071, USA).

### Chronic Social Defeat Stress (CSDS) Model

CSDS was induced in mice as previously described (Golden et al. 2011). First, CD-1 mice were screened for aggressive behavior during social interactions for three consecutive days before the start of the social defeat sessions. They were then housed in the social defeat cage (26.7 cm width × 48.3 cm depth × 15.2 cm height) for 24 h before the start of the defeat interactions on one side of a clear perforated Plexiglas divider (0.6 cm width × 45.7 cm depth × 15.2 cm height). Experimental C57BL/6 J mice were subjected to physical interactions with a novel CD-1 mouse for 10 min once per day over 10 consecutive days. After the interactions, the experimental C57BL/6 J mice were transferred to the opposite side of the social defeat cage and allowed sensory contact over the subsequent 24 h period. Unstressed control C57BL/6 J and CD-1 mice were individually placed in the same cages and rotated daily in a similar manner without exposure to the resident CD-1 mice. After the last interaction, all experimental C57BL/6 J and CD-1 mice were individually housed for 24 h before behavioral testing.

### Social Interaction Test (SIT)

First, C57BL/6 J mice were habituated to the testing suite for 1 h before testing. Second, the mice were placed in a square open-field arena (50 cm × 50 cm × 50 cm), and a small plastic cage was placed in the middle of one side of the square for 2.5 min. The movements of the mice were monitored and recorded automatically by a camera (Sony, SNC-VB600B, Japan) and data analysis software (Panlab, SMART V3.0, Spain); these movements were used as baseline exploratory behavior and locomotion in the absence of a social target (CD-1 mouse). At the end of 2.5 min, the mouse was removed and returned to its home cage until the next stage, and the arena was wiped with 75% alcohol to remove any odors. Third, the movements of the mice in the presence of a novel social target inside the small cage were monitored and recorded for 2.5 min. Fourth, the overall locomotion and the time spent in the interaction were compared between the two recordings. The social interaction ratio was calculated by dividing the time spent in the interaction zone in the presence of the target CD-1 mouse by the time spent in the interaction zone in the absence of the target CD-1 mouse.

### Sucrose Preference Test (SPT)

The SPT reflects anhedonia, a core symptom of depression. On the first day, the water bottle on the home cage was replaced by two 50 ml tubes with sipper tops filled with water, and the mice were allowed 24 h of acclimation to the tubes before the start of testing. On the second day, the water in one of the two tubes was replaced with a 1% sucrose solution. The placement of the two tubes was changed every 8 h to avoid the effects of position preference. On the third day, the food and the two tubes were removed for 24 h to induce thirst. On the fourth day, the food and the two tubes—one filled with water and the other with 1% sucrose solution—were replaced. Both tubes were weighed, and the mice were allowed to drink ad libitum for 12 h. During the tests, the placement of the two tubes was switched 2 times. At the end of the testing, sucrose preference was calculated by dividing the total amount of sucrose consumed by the total amount of fluid consumed over the 12 h of sucrose availability.

### Tail Suspension Test (TST)

The depressive behavior of the mice was analyzed with the TST. Mice were suspended individually by adhesive tape from a tail suspension experimental shelf. The mice were isolated from each other. The tape was placed 1 cm from the tip of the tail. The mice were observed for a period of 3.5 min, and their activity was monitored and recorded automatically by a camera and SMART V3.0 software. The immobility time, defined as the time spent completely motionless, was recorded.

### Microdialysis

A microdialysis probe (4-mm guide cannula length, 0.22-mm membrane outer diameter, 1-mm membrane length, MW cutoff of 50 kD; Eicom Corp) was stereotaxically inserted into the mPFC (15° angle, 1.75 mm anterior and 0.75 mm lateral from bregma, and 1.5 mm ventral to the dura) through the cannula guide. Artificial cerebrospinal fluid (ACSF) (124 mM NaCl; 4.4 mM KCl; 2 mM CaCl_2_; 2 mM MgSO_4_; 25 mM NaHCO_3_; 1 mM KH_2_PO_4_; and 10 mM glucose; pH 7.4) was perfused at a flow rate of 1 μl/min using a microinjection pump. After 1 h of equilibrium, the mouse brain interstitial fluid was continuously collected into microvials for 4 h, and these interstitial fluid samples were subsequently lyophilized and redissolved in 20 μl of ACSF.

### Metabonomic Analysis

Each sample (100 mg) was homogenized in 300 µl of double-distilled water. Cold steel balls were placed in the mixture, which was then incubated on ice for 10 min. The steel ball was removed, 500 µl of pure methanol was added, and the mixture was vortexed at 2500 rpm for 5 min. Next, the mixture was centrifuged at 12,000 rpm at 4 °C for 10 min, and 600 µl of the supernatant was transferred to another centrifuge tube. Then, 100 µl of 5% methanol (95% water) was added to the dried product, and the sample was mixed and centrifuged at 12,000 rpm at 4 °C for 10 min. The supernatant was collected for LC–MS/MS analysis. The analytical conditions were as follows: ultra-performance liquid chromatography (UPLC): column, Waters ACQUITY UPLC HSS T3 C18 (1.8 μm, 2.1 mm*100 mm); column temperature, 35 °C; flow rate, 0.3 ml/min; injection volume, 1 μl; solvent system, water (0.1% formic acid):acetonitrile (0.1% formic acid); gradient program, 95:5 V/V at 0 min, 10:90 V/V at 11.0 min, 10:90 V/V at 12.0 min, 95:5 V/V at 12.1 min, 95:5 V/V at 14.0 min. The original data file generated by LC–MS analysis was converted into mzML format by ProteoWizard software. Peak extraction, alignment, and retention time correction were performed by the XCMS program. The “SVR” method was used to correct the peak area. The peaks were filtered at a deletion rate > 50% in each group of samples. Next, metabolic identification information was obtained by searching the laboratory’s custom-built database and integrating a public database and metDNA. Finally, statistical analysis was carried out with the R program.

### Immunoblotting

The mPFC samples were collected and lysed in RIPA buffer with protease and phosphatase inhibitors (Roche). Protein levels were assessed with a Bradford assay with BSA as the standard. Approximately 10 µg of denatured protein was separated by 10% SDS–polyacrylamide gel electrophoresis and then transferred onto polyvinylidene difluoride (PVDF) membranes (Roche). Nonspecific binding was blocked with TBST (TBS-1% Tween 20) with 5% (w/v) nonfat milk for 2 h at room temperature. The PVDF membranes were then incubated overnight at 4 °C in TBST rabbit anti-GluN2A (1:1000, Cell Signaling Technology, #4205, RRID: AB_2112295) and rabbit anti-GluN2B (1:1000, Cell Signaling Technology, #4207, RRID: AB_1264223) antibodies, which have been described in previous studies (Taniguchi et al. 2009; Unsicker et al. 2021). To examine GluA1, GluA2, syntaxin 1A, and PSD95 expression, Western blot analysis was performed as described in a previous study (Zhou et al. 2020; Borland et al. 2020; Zhang et al. 2021; Huang et al. 2021). Western blots were performed with rabbit anti-GluA1 (1:1000, Cell Signaling Technology, #13185, RRID: AB_2732897), rabbit anti-GluA2 (1:1000, Cell Signaling Technology, #5306, RRID: AB_10622024), rabbit anti-syntaxin 1A (1:1000, Cell Signaling Technology, #18572, RRID: AB_2798803), and rabbit anti-PSD95 (1:1000, Abcam, ab18258, RRID: AB_444362) antibodies. To examine CDO1, CSAD, and TAUT expression, Western blot analysis was performed as described in a previous study (Geillinger et al. 2014; Guerra et al. 2021). Western blotting was performed with rabbit anti-CDO1 (1:1000, Proteintech, 12589–1-AP, RRID: AB_10638145), rabbit anti-CSAD (1:1000, Abcam, ab91016, RRID: AB_10713222), rabbit anti-TAUT (1:1000, Invitrogen, #TG2607332, RRID: AB_2736681), and mouse anti-β-actin (1:10000, Proteintech, 60008–1-ig, RRID: AB_2289225) antibodies. The membranes were then incubated at room temperature for 2 h in TBST with the corresponding secondary antibodies: goat anti-rabbit IgG (H&L) (1:10000, Abcam, ab205718, RRID: AB_2819160) or goat anti-mouse IgG (H&L) (1:10000, Abcam, ab205719, RRID: AB_2755049). Protein bands were detected by chemiluminescence (Tanon, 5200 Multi, China) and quantified by densitometry with ImageJ 7.0. Protein levels were normalized to that of β-actin as a control.

### Immunohistochemistry

Mice were anesthetized with 5% pentobarbital and transcardially perfused with 20 ml of ice-cold PBS followed by 40 ml of 4% paraformaldehyde (PFA). Brain samples were postfixed in 4% PFA for 2 h, followed by an additional 48 h of dehydration in 30% sucrose at 4 °C. Then, the brain samples were sectioned at a thickness of 18 μm using a Cryostat (Leica, CM-1950, Germany) at − 20 °C. The sections were washed with 0.01 mM PBS (pH 7.4) and blocked with 3% bovine serum albumin (3% BSA and 0.3% Triton-X in PBS) for 1 h at room temperature, followed by overnight incubation at 4 °C with the following primary antibodies: anti-taurine (1:200, Millipore, AB5022, RRID: AB_91642) and anti-GluN2A (1:500, Millipore, AB1555P, RRID: AB_90770). Both of these antibodies have been described in previous studies (Nivison-Smith et al. 2013; Downie et al. 2010; King et al. 2006). After rinsing with PBS, the sections were incubated with the following secondary antibody conjugated with a fluorochrome for 2 h in PBS: Alexa 594-AffiniPure donkey anti-rabbit IgG antibody (1:500, Invitrogen, A21207, RRID: AB_141637). Next, the sections were incubated with Hoechst (1:1000) for another 10 min and then washed with 0.01 mM PBS. Finally, the sections were mounted and cover slipped with Fluoromount-G and stored at − 20 °C. A confocal laser scanning microscope (Olympus, FV1000, Japan) and confocal software (Olympus, Fluoview Ver4.2b, Japan) were used for image acquisition. Briefly, the slides were scanned under a laser confocal microscope at excitation wavelengths of 405 nm and 543 nm and emission wavelengths of 450 nm and 590 nm. The parameters were set as follows: objective lens (20 × , numerical aperture = 0.75), sequential (line), and pixel (1024 * 1024). All images were captured in a dark room at a temperature of 25 °C. FLUOVIEW and ImageJ software were used for image analysis.

### Golgi-Cox Staining

The mouse brains were rinsed in double-distilled water, immersed in impregnation solution made by mixing equal volumes of commercial solutions (potassium dichromate, mercuric chloride, and potassium chromate) and stored for 1 week in the dark at room temperature. The blocks were then transferred into PBS. Subsequently, coronal sections were cut at a thickness of 150 µm in the dark using a vibratome and mounted on gelatinized slides in PBS. The slides were rinsed in double-distilled water, stained with the staining solution, dehydrated in successive ethanol baths, cleared in xylene, and cover slipped with Permount TM mounting medium. A confocal microscope and FLUOVIEW software were used for image acquisition. Images were acquired at a resolution of 2048 pixels in the X–Y–Z dimensions, with the Z dimension being variable. For the analysis of dendritic branches, neurons were imaged using a 20 × objective lens (na = 0.75). The Z-dimensional increment was 2 µm. For the analysis of spines, neurons were imaged using a 60 × objective lens (na = 1.42). The Z-dimensional increment was 0.6 µm. Neurons were randomly selected for analysis from at least 10 brain slices of three control, three CSDS, and three taurine-treated CSDS mice. The stack images were analyzed using Imaris software (version 7.7.1, serial number: 32 mr-rfhf-7 hbu-jb58, Bitplane, Switzerland). The total length of each dendrite and the cell body volume were automatically calculated. For Sholl analysis, spheres were constructed continuously from the center of the cell body with radial increments of 10 µm. The number of intersections between each sphere and the dendrites was calculated for comparison. Compared with the use of 2D images, the use of 3D images for Sholl analysis can provide statistical results that are closer to the actual structure of the neurons, especially when analyzing dendrites with different angles. Spine shape was defined by the length of the spine and the widths of the spine neck and spine head, which allowed us to classify the spines into four types: stubby, mushroom, long thin, and filopodia. The stubby type had a length < 1 µm; the mushroom type had a length > 3 µm, and the maximum width of the head/the mean width of the neck was > 2; the long thin type had a ratio of the mean width of the head/mean width of the neck ≥ 1; and the rest of the spines were classified as filopodia. Spine measurements were performed using a MATLAB-X Tension Spines Classifier in Imaris. All imaging data were analyzed by an investigator who was blinded to the experimental groups.

### Chronic Restraint Stress (CRS) Mice

The mice subjected to the CRS procedure were placed into a 50 ml centrifuge tube for 4 h (10:00 a.m.–2:00 p.m.) once a day for 14 days, with food and water fasting during the restraint period. Behavioral tests were started 1 h after the 4 h period of restraint stress on the 14th day. The mice in the control group stayed in their home cages until the behavior test started.

### Statistical Analysis

Mice were randomly assigned to the control, CSDS, and taurine-treated CSDS mouse groups. Analyses were performed in a manner that was double blinded to the treatment assignments in all experiments. Statistical analyses were performed using GraphPad Prism software v7.0, and all data are presented as the mean ± SD. Statistical significance was evaluated using Student’s *t* test or one-way ANOVA followed by the Tukey–Kramer post hoc test. *P* < 0.05 was used to determine significance where indicated.

## Results

### Taurine Deficiency in mPFC Interstitial Fluid in CSDS Mice

**Fig. 1:**
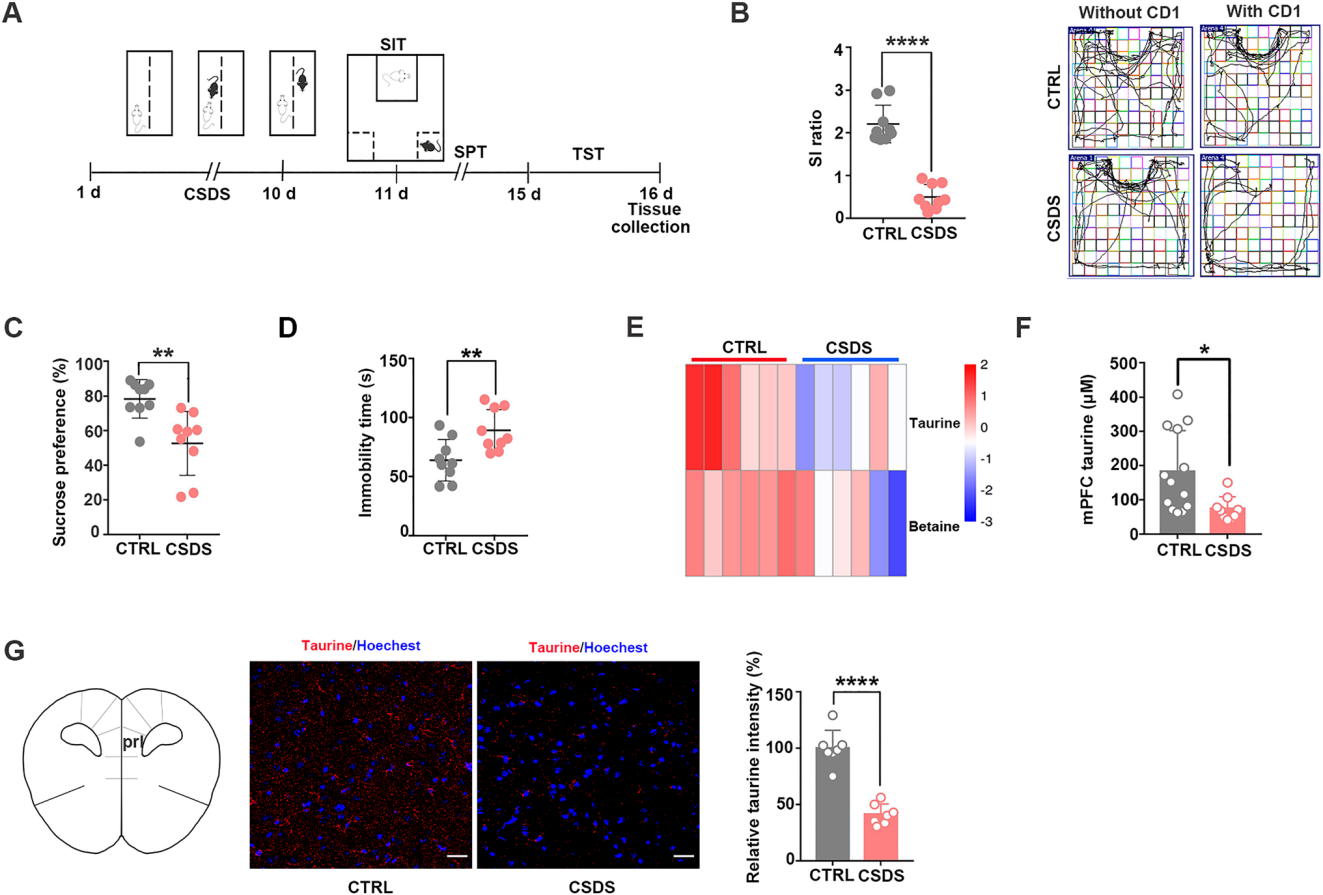
Decreased taurine levels in the mPFC of CSDS mice. **A** Experimental timeline of the 10-day CSDS paradigm; SIT, SPT, and TST behavioral screening; and mPFC tissue collection. **B** CSDS induced depression-like behaviors as assessed by social interaction tests (*n* = 9 per group; Student’s *t* test, ****p < 0.0001). **C** CSDS induced depression-like behaviors as assessed by the sucrose preference test (*n* = 9 per group; Student’s *t* test, **p < 0.01). **D** CSDS induced depression-like behaviors as assessed by the tail suspension test (*n* = 9 per group; Student’s *t* test, **p < 0.01). **E** Broad-spectrum metabolite analyses showed that the levels of taurine and betaine were significantly decreased in the mPFC interstitial fluid in CSDS mice (*n* = 6 per group). **F** The taurine levels were reduced in the mPFC of CSDS mice after 10 days of CSDS as determined by a taurine test kit (*n* = 9–13 per group; Student’s *t* test, *p < 0.05). **G** The fluorescence intensity derived from taurine was decreased in the mPFC of CSDS mice after 10 days of CSDS (*n* = 7 per group; Student’s *t* test, ****p < 0.0001, scale bars = 50 μm). The data are presented as the mean ± SD

Chronic stress-associated neurometabolic abnormalities are thought to be critical for the development of MDD (Huang et al. 2020; Ernst et al. 2017). To identify the critical metabolic substances that are involved in chronic stress-induced depression, we used CSDS mice to mimic depression-like behaviors in humans. The CSDS mice showed depressive symptoms, which were validated by the SIT, SPT, and TST (Fig. 1A). In the SIT, CSDS mice exhibited a significantly lower SI ratio and sparser trajectory near the aggressor (CD1 mice) than the control mice (Fig. 1B). In addition, the sucrose preference was decreased (Fig. 1C), and the immobility time was increased (Fig. 1D) in the CSDS mice.

Next, we screened for aberrant metabolic substances in the mPFC, the essential brain area for MDD (Price and Duman 2020), in CSDS mice by metabonomic analysis. We found significant reductions in the taurine and betaine levels in the mPFC interstitial fluid of CSDS mice compared with control mice as determined by microdialysis (Fig. 1E). We found that taurine content was markedly decreased in the mPFC of CSDS mice by using a Taurine Assay Kit (Fig. 1F). Immunohistochemistry further confirmed that taurine was markedly decreased in the mPFC of CSDS mice (Fig. 1G). We additionally investigated the levels of interstitial taurine in chronic restraint stress (CRS) mice, another depressive animal model. After 14 days of CRS treatment, the mice showed typical depression-like behaviors, including decreased sucrose preference and increased immobility time (Supplementary Fig. 1A, B). The levels of interstitial taurine in the mPFC were also significantly decreased in the CRS mice (Supplementary Fig. 1C).

To explore whether taurine deficit is associated with impaired synthesis or absorption in CSDS mice, we measured the expression of enzymes involved in taurine synthesis, including cysteine dioxygenase (CDO) and cysteine-sulfinate decarboxylase (CSAD). We found that the expression of CDO and CSAD was not changed in the mPFC of CSDS mice. In addition, the expression of TAUT, a taurine transporter, remained unchanged (Supplementary Fig. 2).

Next, to determine whether a taurine deficit is critical for the development of depression, we used a structural analog of taurine, β-alanine, to competitively inhibit the uptake of taurine. Mice injected with β-alanine exhibited a decreased SI ratio (Supplementary Fig. 3A). However, the level of taurine in the mPFC was not affected after β-alanine administration (Supplementary Fig. 3B). Altogether, these findings suggest that taurine dysfunction in the brain promotes depression-like phenotypes in mice.

### Taurine Administration Alleviates Depression-Like Behaviors in CSDS Mice

**Fig. 2:**
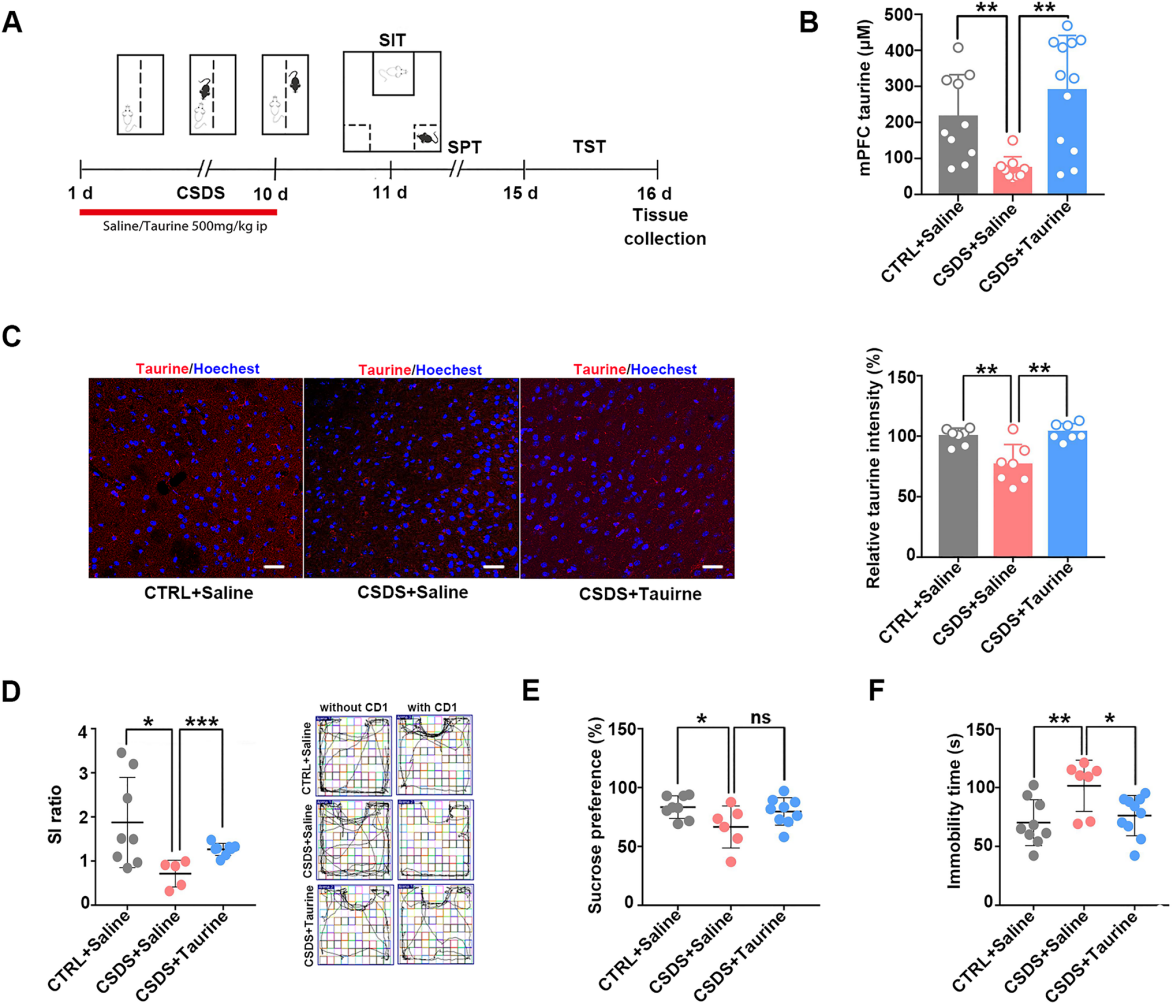
Taurine administration alleviated depression-like behaviors in CSDS mice. **A** Experimental timeline of the intraperitoneal (i.p.) injection of taurine and behavioral studies. **B** Taurine levels were reduced in the mPFC of CSDS mice after 10 days of CSDS as determined by a taurine test kit. Taurine levels were increased in the mPFC after the i.p. injection of taurine (*n* = 9–12 per group; one-way ANOVA, **p < 0.01). **C** The fluorescence intensity derived from taurine was decreased in the mPFC of CSDS mice after 10 days of CSDS. The fluorescence intensity derived from taurine in the mPFC was increased after the i.p. injection of taurine (*n* = 7 per group; one-way ANOVA, **p < 0.01, scale bars = 50 μm). **D** Taurine treatment partially rescued social avoidance in CSDS mice (*n* = 5–8 per group; one-way ANOVA, *p < 0.05, ***p < 0.001). **E** There was no effect on sucrose preference after taurine treatment (*n* = 6–9 per group; one-way ANOVA, *p < 0.05). **F** Taurine treatment rescued the immobility time in CSDS mice (*n* = 7–10 per group; one-way ANOVA, *p < 0.05, **p < 0.01). The data are presented as the mean ± SD

To further assess whether taurine supplementation can rescue abnormal behaviors in CSDS mice, we administered exogenous taurine to CSDS mice via intraperitoneal injection (i.p.) for 10 days during the CSDS period (Fig. 2A). Taurine deficits in CSDS mice were effectively restored by intraperitoneal injection (Fig. 2B, C). Notably, taurine treatment partially rescued social avoidance in CSDS mice (Fig. 2D). However, there was no effect on sucrose preference (Fig. 2E). Immobility time was also significantly rescued by taurine treatment (Fig. 2F). These results indicate that taurine supplementation significantly alleviates depression-like behaviors in CSDS mice.

### Taurine Supplementation Rescues Dendritic Structural Impairment in CSDS Mice

**Fig. 3:**
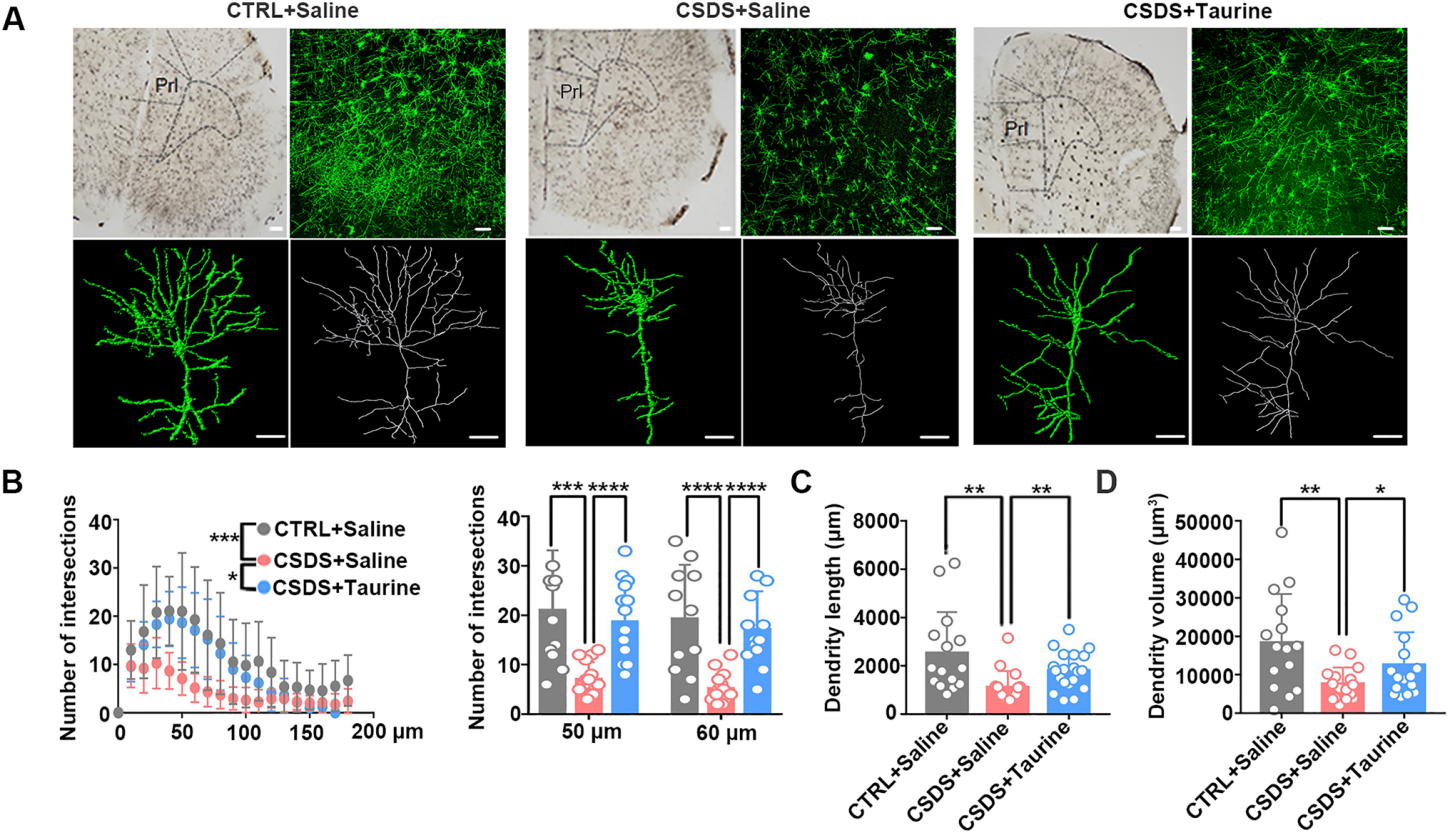
Taurine treatment prevented the reduction in dendritic complexity in the mPFC. **A** Representative images showing the distributions of neurons in the mPFC of control, CSDS, and taurine-treated CSDS mice. Top, scale bar: 100 µm. Bottom, scale bar: 30 µm. **B** Sholl analysis showed reduced dendritic complexity of neurons in CSDS mice compared with control mice and taurine-treated CSDS mice, and the Sholl radius significantly differed among the groups (*n* = 15 per group; Friedman’s *M* test, *p < 0.05, ***p < 0.001; Sholl radius in 50 µm, one-way ANOVA, ***p < 0.001; Sholl radius in 60 µm, one-way ANOVA, ****p < 0.0001).** C** The dendritic length of neurons was decreased in CSDS mice compared with control mice. The dendritic length of neurons was decreased in CSDS mice compared with taurine-treated CSDS mice (*n* = 15 per group; one-way ANOVA, **p < 0.01). **D** The dendritic volume of neurons was decreased in CSDS mice compared with control mice. The dendritic volume of neurons was decreased in CSDS mice compared with taurine-treated CSDS mice (*n* = 15 per group; one-way ANOVA, *p < 0.05, **p < 0.01). The data are presented as the mean ± SD

The function of taurine is important (Park et al. 2019b), as it provides neurons with nutritional support and is fundamental for the functional regulation of dendritic spines (Jangra et al. 2020; Xiao et al. 2015). Here, we performed 3D reconstruction of pyramidal neurons in the mPFC and analyzed the complexity of their dendrites (Fig. 3A). We found that the detailed dendritic processes of these pyramidal neurons were conspicuously divergent between control and CSDS mice. The number of dendritic intersections was reduced in the mPFC of CSDS mice, especially in dendrites that were 50 µm and 60 µm in length, and taurine supplementation significantly alleviated these reductions (Fig. 3B). Moreover, both the length and volume of dendrites were decreased in CSDS mice, and taurine treatment rescued these reductions (Fig. 3C, D). Altogether, our findings indicated that dendritic complexity was impaired and that taurine supplementation protected mPFC dendrites from damage in CSDS mice.

**Fig. 4:**
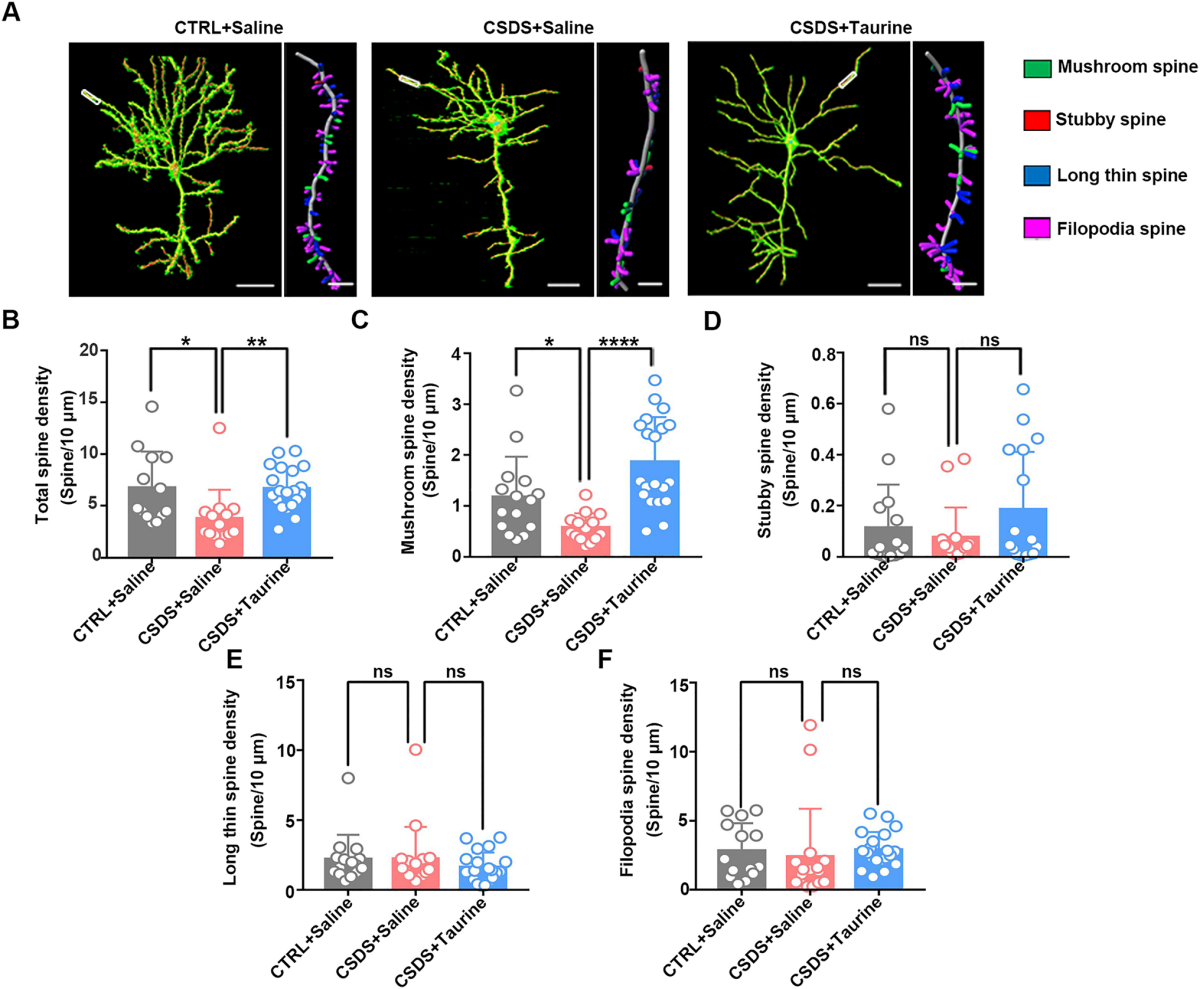
Taurine supplementation rescued dendritic spine impairment. **A** Representative spine and reconstructed images of the mPFC in the three groups of mice. Left, scale bar: 100 µm. Right, scale bar: 5 µm. **B** The total dendritic spine density was reduced in CSDS mice compared with control mice. The total dendritic spine density was increased in taurine-treated CSDS mice compared with CSDS mice (*n* = 15 per group; one-way ANOVA, *p < 0.05, **p < 0.01). **C** The density of mushroom spines was reduced in CSDS mice compared with control mice. The density of mushroom spines was increased in taurine-treated CSDS mice compared with CSDS mice (*n* = 15 per group; one-way ANOVA, *p < 0.05, ****p < 0.0001). **D** The densities of stubby spines did not obviously differ in CSDS and taurine-treated CSDS mice compared with control mice (*n* = 15 per group; one-way ANOVA, *p* > 0.05). **E** The densities of long thin spines did not obviously differ in CSDS and taurine-treated CSDS mice compared with control mice (*n* = 15 per group; one-way ANOVA, *p* > 0.05). **F** The densities of filopodia did not obviously differ in CSDS and taurine-treated CSDS mice compared with control mice (*n* = 15 per group; one-way ANOVA, *p* > 0.05). The data are presented as the mean ± SD

We further analyzed the dendritic spine morphologies (Fig. 4A). The total dendritic spine and mushroom spine (mature spine) densities were significantly decreased in CSDS mice and were rescued after exogenous taurine administration (Fig. 4B, C). However, the densities of other spine types, including the stubby, long thin, and filopodia spine types, did not differ markedly between control mice and CSDS mice with or without taurine treatment (Fig. 4D–F). These data suggest that the taurine deficits caused by CSDS are involved in abnormal dendritic and spine morphology and that taurine supplementation can alleviate dendrite and spine loss.

### Taurine Treatment Rescues the Expression of GluN2A and Syntaxin 1A in Mice Exhibiting Depression-Like Behaviors

**Fig. 5:**
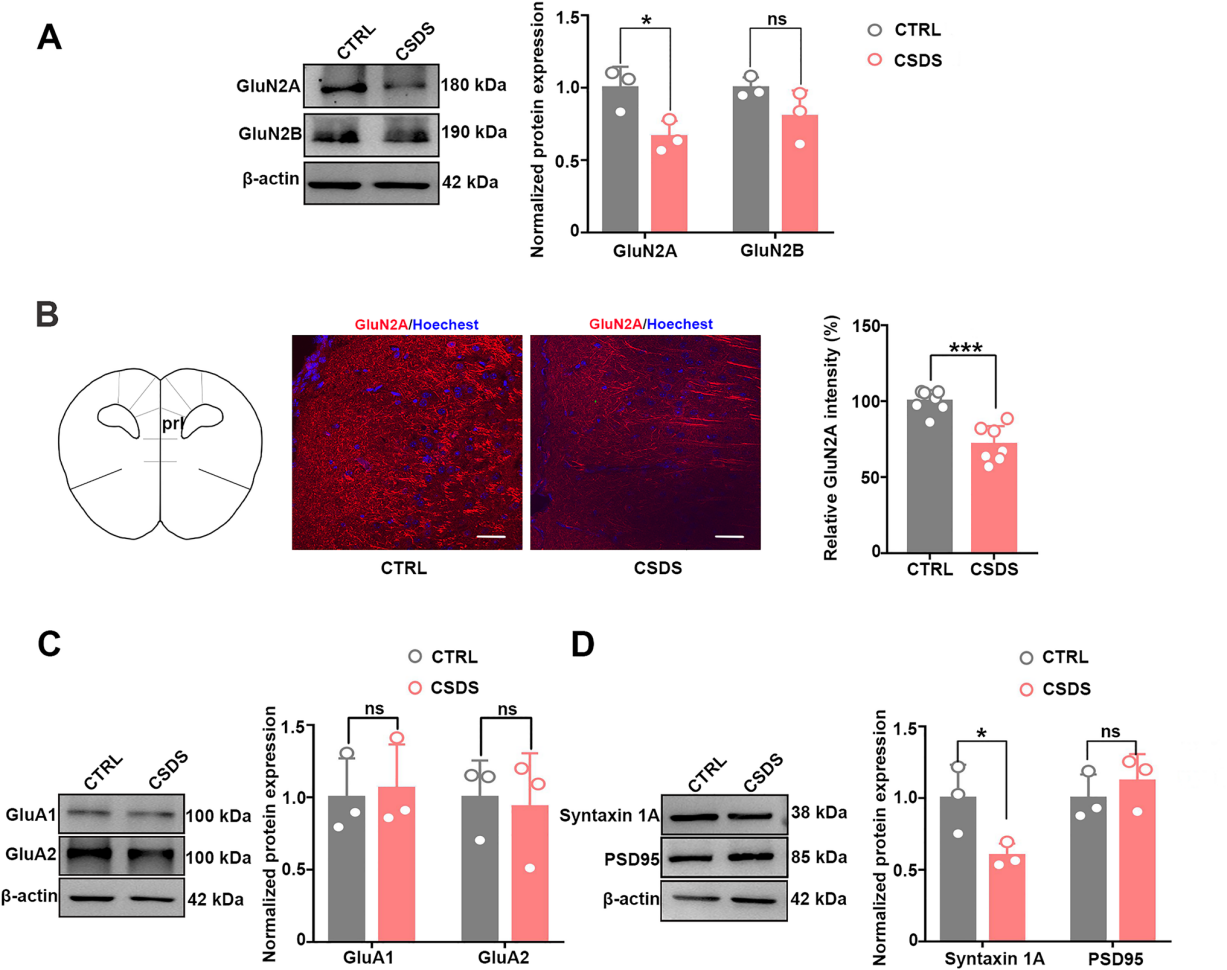
The expression levels of proteins associated with taurine transfer and synaptic transmission in CSDS mice. **A** The protein levels of GluN2A and GluN2B in control and CSDS mice (*n* = 3 per group; Student’s *t* test, *p < 0.05).** B** The fluorescence intensity derived from GluN2A was decreased in the mPFC of the mice after 10 days of CSDS (*n* = 7 per group; Student’s *t* test, ***p < 0.001, scale bars = 50 μm).** C** The protein levels of GluA1 and GluA2 in control and CSDS mice (*n* = 3 per group; Student’s *t* test, *p* > 0.05).** D** The protein levels of syntaxin 1A and PSD95 in control and CSDS mice (*n* = 3 per group; Student’s *t* test, *p < 0.05). The data are presented as the mean ± SD

N-methyl-d-aspartate receptors (NMDARs) are speculated to be involved in the pathogenesis of several neurological diseases, such as depression (Murrough et al. 2017). Taurine exerts its protective function against glutamate-induced neuronal excitotoxicity by reducing the glutamate-induced elevation of intracellular free calcium levels (Wu et al. 2005). However, whether glutamate signals and synaptic proteins are associated with taurine-mediated neural protection is still unclear. Therefore, we first examined the protein levels of NMDA and alpha-amino-3-hydroxy-5-methyl-4-isoxazolepropionic acid (AMPA) receptors, two main types of glutamate receptors. We observed a deficit in GluN2A but not GluN2B, two subtypes of NMDA receptors, in the mPFC of CSDS mice (Fig. 5A, B). However, no significant differences were found in GluA1 and GluA2, two subtypes of AMPA receptors (Fig. 5C). In addition, we examined the levels of the synapse-associated proteins syntaxin 1A and PSD95, which are mainly located in the presynaptic and postsynaptic membranes, respectively. We found a loss of syntaxin 1A but not PSD95 in CSDS mice (Fig. 5D). These data indicated that impaired GluN2A and syntaxin 1A expression occurred simultaneously with taurine deficits in CSDS mice.

**Fig. 6:**
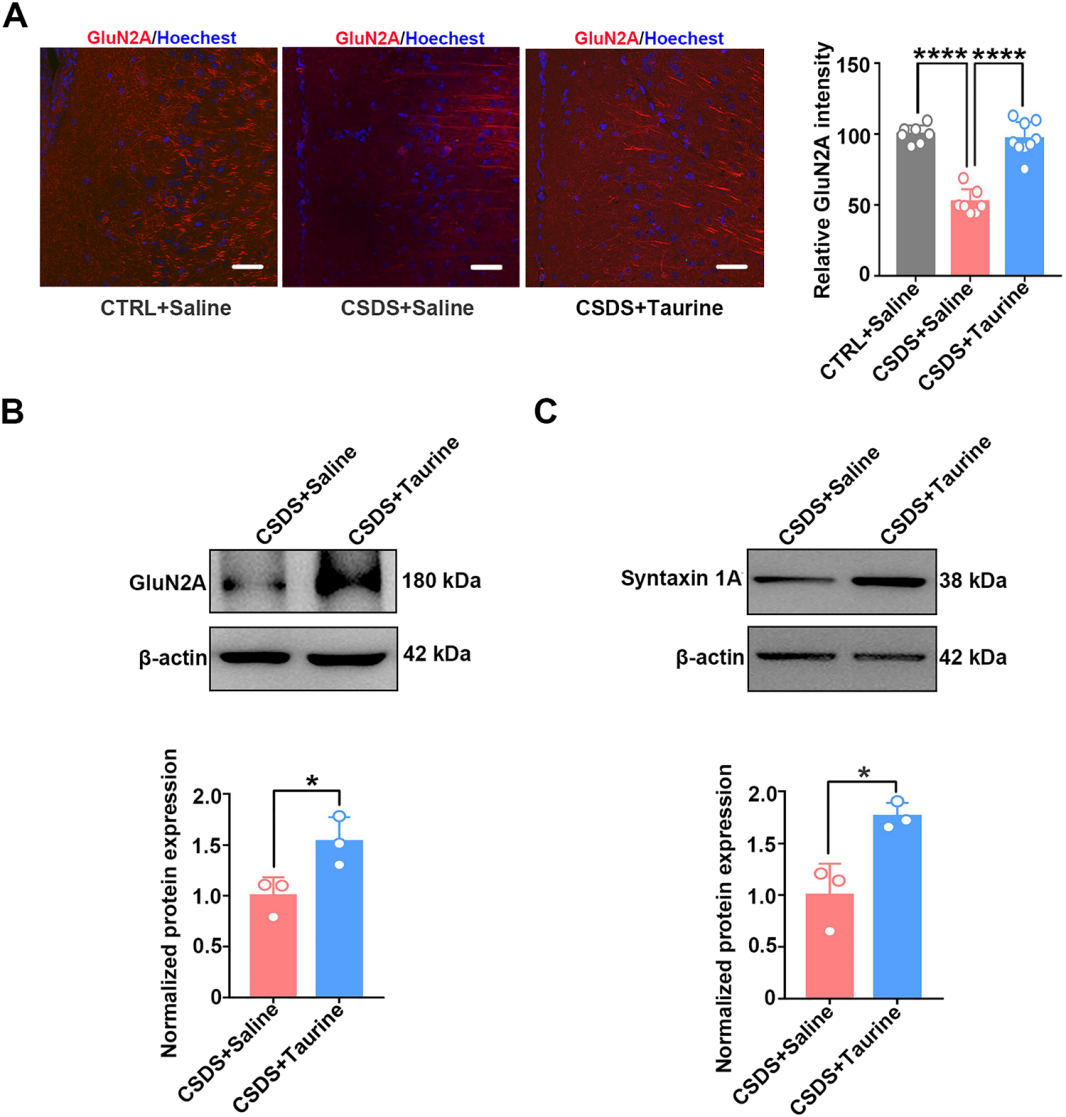
Taurine administration protected against decreased GluN2A expression in CSDS mice. **A** The fluorescence intensity derived from GluN2A was increased in the mPFC of taurine-treated CSDS mice (*n* = 7 per group; one-way ANOVA, ****p < 0.0001, scale bars = 50 μm). **B** The protein levels of GluN2A in CSDS and taurine-treated CSDS mice (*n* = 3 per group; Student’s *t* test, *p < 0.05).** C** The protein levels of syntaxin 1A in CSDS and taurine-treated CSDS mice (*n* = 3 per group; Student’s *t test*, *p < 0.05). The data are presented as the mean ± SD

We performed immunoblotting and immunohistochemistry analyses to measure the expression of GluN2A to explore whether the decrease in synaptic proteins could be reversed by taurine administration. The expression of GluN2A was recovered in taurine-treated CSDS mice compared with saline-treated CSDS mice (Fig. 6A, B). In addition, the expression of syntaxin 1A was rescued in taurine-treated CSDS mice compared with saline-treated CSDS mice (Fig. 6C). Taken together, these data suggest that the level of taurine associated with GluN2A and syntaxin 1A expression is involved in the development of depression.

## Discussion

In this study, we showed the function and mechanism of taurine in preventing the development of depression under chronic stress. Importantly, we found that exogenous taurine supplementation alleviated depression-like behaviors, rescued impaired dendritic structures, and restored synaptic protein expression in CSDS mice.

The pathogenesis of depression is complicated and involves both neuroendocrine and central nervous system dysfunction as well as neurobiological and morphological alterations across several brain regions that are particularly vulnerable to stress (Tse et al. 2014; Castaneda et al. 2015). Although many types of antidepressant medications have beneficial effects on patients, most of the currently used tricyclic antidepressants, monoamine oxidase inhibitors, and selective serotonin reuptake inhibitors have been shown to have various disadvantages, including slow onset, low response rates, toxic effects to organs, and drug resistance (Nemeroff 2007; Penn and Tracy 2012). Therefore, ingredients from natural foods that could prevent depression and medicine with fewer adverse effects are being increasingly welcomed (Wu et al. 2017). As a semiessential amino acid for humans, taurine has an observed safe level of supplemental intake in normal healthy adults of up to 3 g/day and was verified to have no adverse effects at up to 1000 mg/kg/day by the European Food Safety Authority (Shao and Hathcock 2008). We chose a dose of 500 mg/kg taurine for the treatment of CSDS mice according to previous reports and our pilot study. We did not perform toxicity assessments of taurine administration in mice. We found that taurine has a very wide safe dosage range for humans, animals, and cells (Chao et al. 2014; Van Hove et al. 2019; Wu et al. 2017).

Evidence has demonstrated that the concentrations of taurine are greatly diminished in the plasma, cerebrospinal fluid, and brains of patients with depression (Caletti et al. 2012; Perry et al. 1975). Taurine pretreatment has been shown to exert antidepressant effects in a rat model of mild stress-induced depression (Wu et al. 2017). Here, we found that the taurine content was decreased in the mPFC, a susceptible brain region that is most frequently identified in depression patients and mouse models, which was consistent with previous clinical data (Pu et al. 2021; Belleau et al. 2019; Yan et al. 2018). However, one report indicated that taurine levels were elevated in depressed patients (Lima et al. 2003). To date, the conclusions on taurine in depression patients and mouse models are inconsistent.

Taurine pretreatment was previously found to prevent neuronal death in the cerebral and cerebellar cortices and abrogate the decrease in dendritic arborization (Owoeye et al. 2018). Here, we found that taurine treatment attenuated the decrease in dendritic length and volume and the impairment of morphological complexity in CSDS mice. Taurine supplementation also prevented decreases in dendritic spine densities, especially the numbers of mushroom spines. The mushroom spines are mature dendritic spines and are the most abundant type (Kaul et al. 2020). These results are consistent with previous reports (Noorafshan et al. 2018). The structures of dendritic spines are closely related to synaptogenesis, and changes in the length and volume of dendritic spines may affect synaptic transmission through the proteins distributed in these synapses. A variety of glutamate receptors and their corresponding scaffolding proteins are usually located on the tips of dendritic spines (Pandian et al. 2020). After blockade of NMDARs, synaptic density did not increase due to the induction of high-frequency stimulation in a depression mouse model (Kennedy et al. 2010; Aarts et al. 2002).

We found that taurine could efficiently rescue the expression of GluN2A, an NMDAR subunit (Ragguett et al. 2019; Amidfar et al. 2019; Gilbert et al. 2018; Ostadhadi et al. 2017), which may be implicated in taurine-mediated neural protection by promoting dendritic growth and increasing the number of dendritic spines. Consistent with NMDARs playing a role in depression, NMDA-induced synaptic depression is associated with reduced synaptic plasticity (Compans et al. 2021). While taurine was previously reported to exert multiple effects on GluN2B, an interaction between taurine and the GluN2A subtype cannot be ruled out (Chan et al. 2015). Moreover, taurine was previously reported to interact with GluN2A (Hansen et al. 2020). We speculate that the taurine-GluN2A interaction is implicated in the alleviation of depression-like behaviors in CSDS mice. Ketamine exerts its rapid antidepressant function via the tonic activation of GluN2B-containing NMDARs (Miller et al. 2014). Therefore, taurine and ketamine may interact with different NMDARs to exert antidepressant effects. In addition, future studies are required to determine the transmission of neurotransmitters in response to taurine. As previously reported, NMDARs are expressed on both presynaptic and postsynaptic membranes (Bardoni et al. 2004; Xie et al. 2022; Baez et al. 2018). Therefore, taurine-associated GluN2A expression changes occurring on presynaptic and/or postsynaptic membranes need to be further investigated through immunoelectromicroscopy and electrophysiological analysis.

Taurine can also improve anxiety-like behaviors in animals (Park et al. 2019a; Kong et al. 2006), and reduced taurine levels have been reported in a population of patients with anxiety (Strasser et al. 2019). However, these studies mainly reported taurine-associated antianxiety effects without further investigating the underlying mechanisms.

This study has several limitations. First, we measured the expression levels of enzymes involved in taurine synthesis, including CDO and CSAD. The activities of these enzymes were not investigated, and the mechanism underlying the decreased taurine levels in CSDS mice was not explained. Second, we found decreases in the levels of GluN2A and syntaxin 1A, but we did not directly rescue the expression of these two proteins in CSDS mice. Finally, we did not deplete taurine in the brains of control mice to investigate the direct effect of taurine deficiency on depression-like behaviors.

In summary, a taurine deficit in the brain is associated with the development of depression in CSDS mice. Taurine supplementation is a promising therapeutic strategy for alleviating depressive symptoms by preventing dendritic structural impairments and synaptic protein deficits.

## Supplementary Information

Below is the link to the electronic supplementary material.

Supplementary file1 (DOCX 4263 kb)

## Data Availability

All original data, antibodies, and custom reagents are available from the corresponding author’s laboratory.
