# Supplementary material for: Taurine Alleviates Chronic Social Defeat Stress-Induced Depression by Protecting Cortical Neurons from Dendritic Spine Loss
Source: Cell Mol Neurobiol. 2022 Apr 18;43(2):827–40. doi: 10.1007/s10571-022-01218-3 (PMC9958166; doi:10.1007/s10571-022-01218-3)
Supplement: Supplementary file 1 — Supplementary file1 (DOCX 4263 kb) [file 10571_2022_1218_MOESM1_ESM.docx]

**
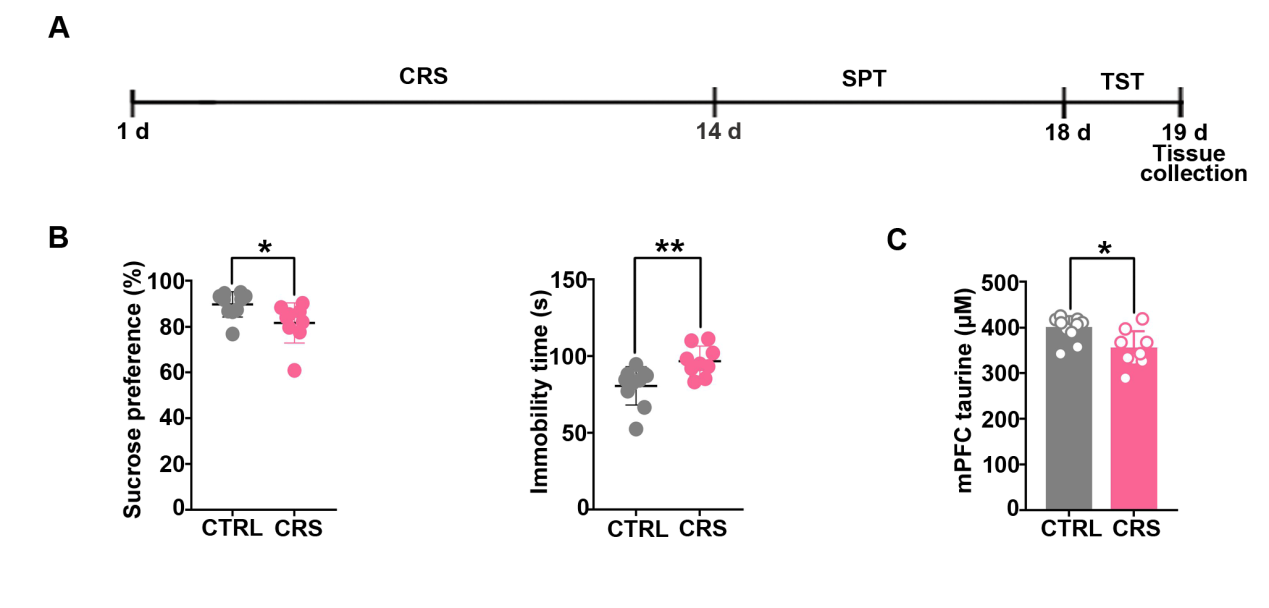
**

**Supplementary Fig. 1 Decreased taurine levels in the mPFC of CRS mice. A** Experimental timeline of the CRS paradigm, SPT and TST behavioral screening, and tissue collection of the mPFC. **B** CRS induced depression-like behaviors as assessed by sucrose preference and tail suspension tests (n = 9 per group, Student’s *t test*, ^*^*p* < 0.05, ^**^*p* < 0.01). **C** Taurine levels were reduced in the mPFC of CRS mice as determined by a taurine test kit (n = 9 per group, Student’s *t test*, ^*^*p* < 0.05). The data are presented as the mean ± SD.


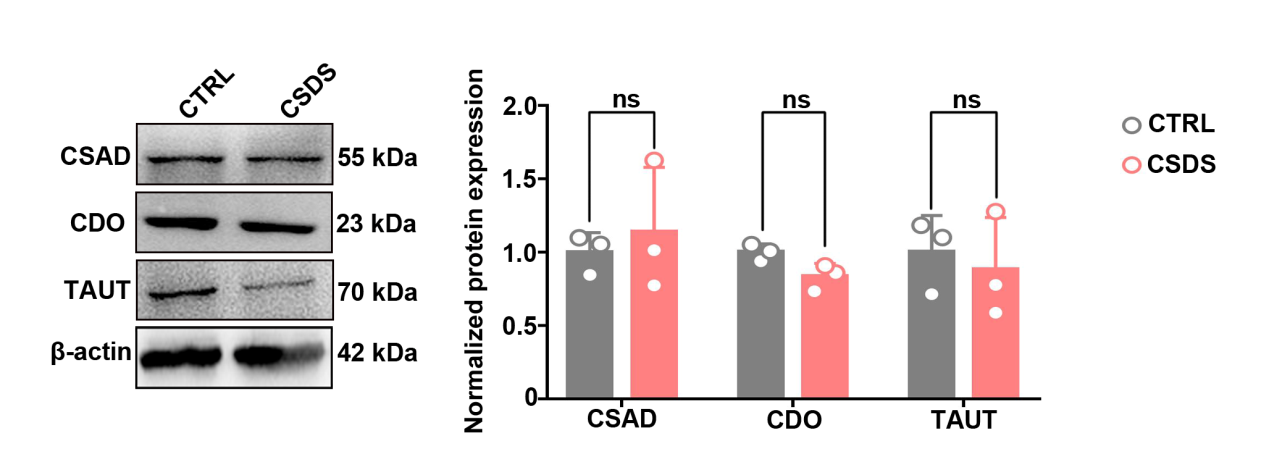


**Supplementary Fig. 2 Expression of enzymes involved in taurine synthesis**. The protein levels of CSAD, CDO and TAUT in the control and CSDS mice (n = 3 biological replicates, Student’s *t test*, *p* > 0.05). The data are presented as the mean ± SD.


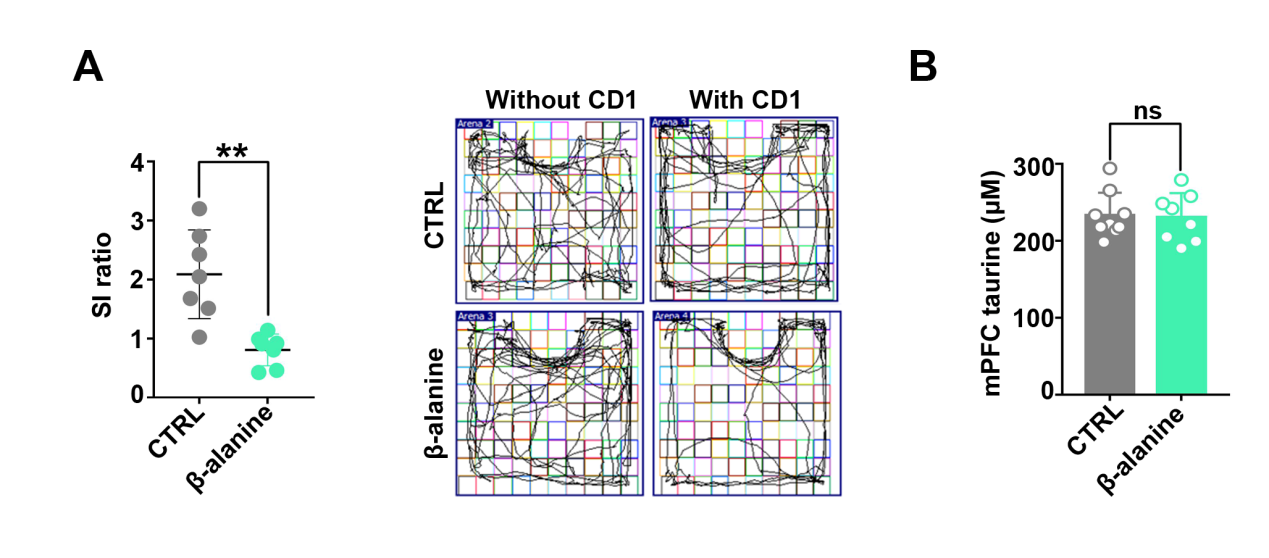


**Supplementary Fig. 3 The taurine inhibitor β-alanine causes depression-like behavior. A** β-Alanine (500 mg/kg, once per day for 10 days) injection induced depression-like behaviors in mice as assessed by social interaction tests (n = 7 per group, Student’s *t test*, ^**^*p* < 0.01). **B** Taurine levels were shown to be unchanged in the mPFC of β-alanine-injected mice as determined by the taurine test kit (n = 8 per group, Student’s *t test*, *p* > 0.05). The data are presented as the mean ± SD.


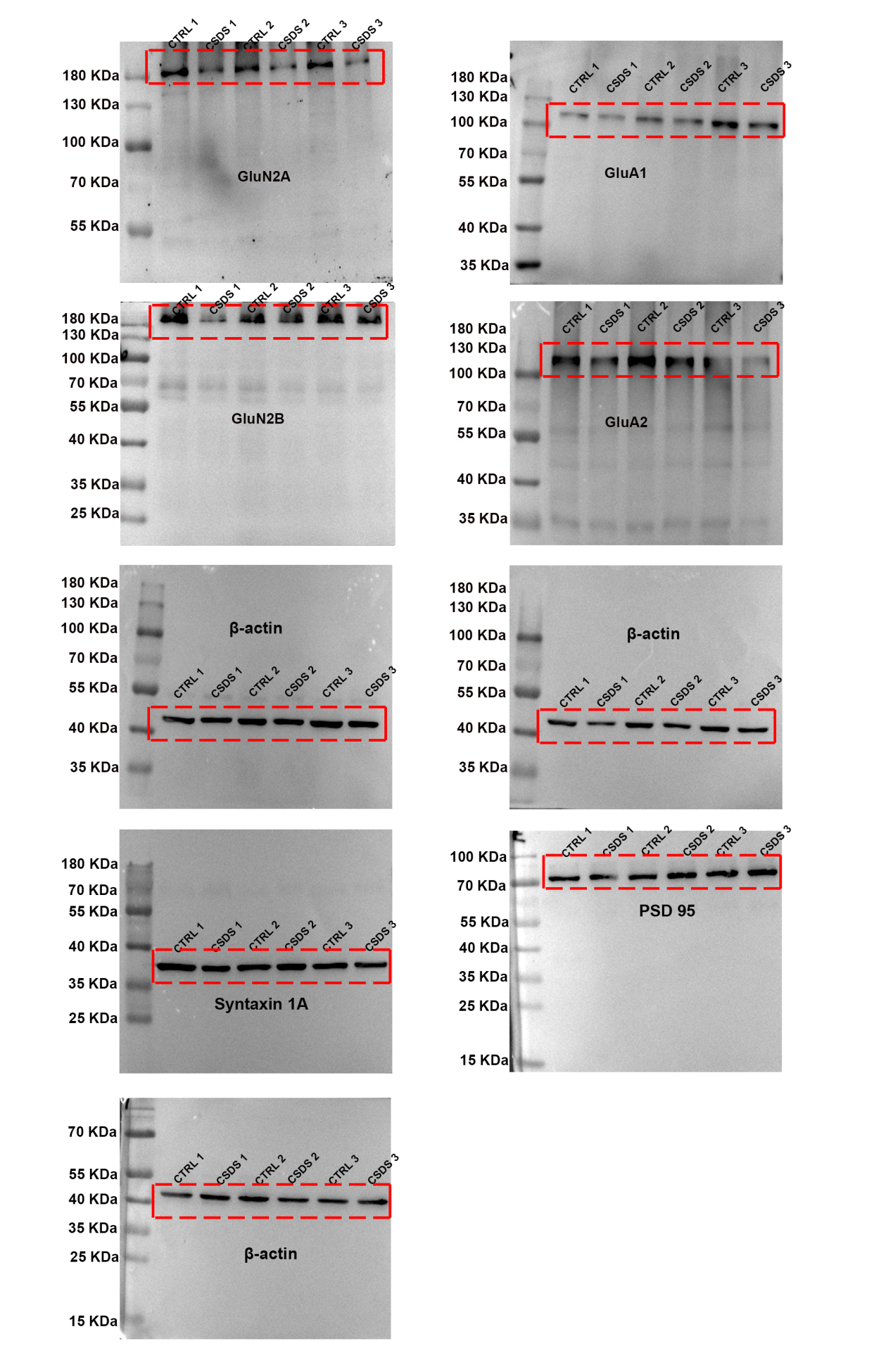


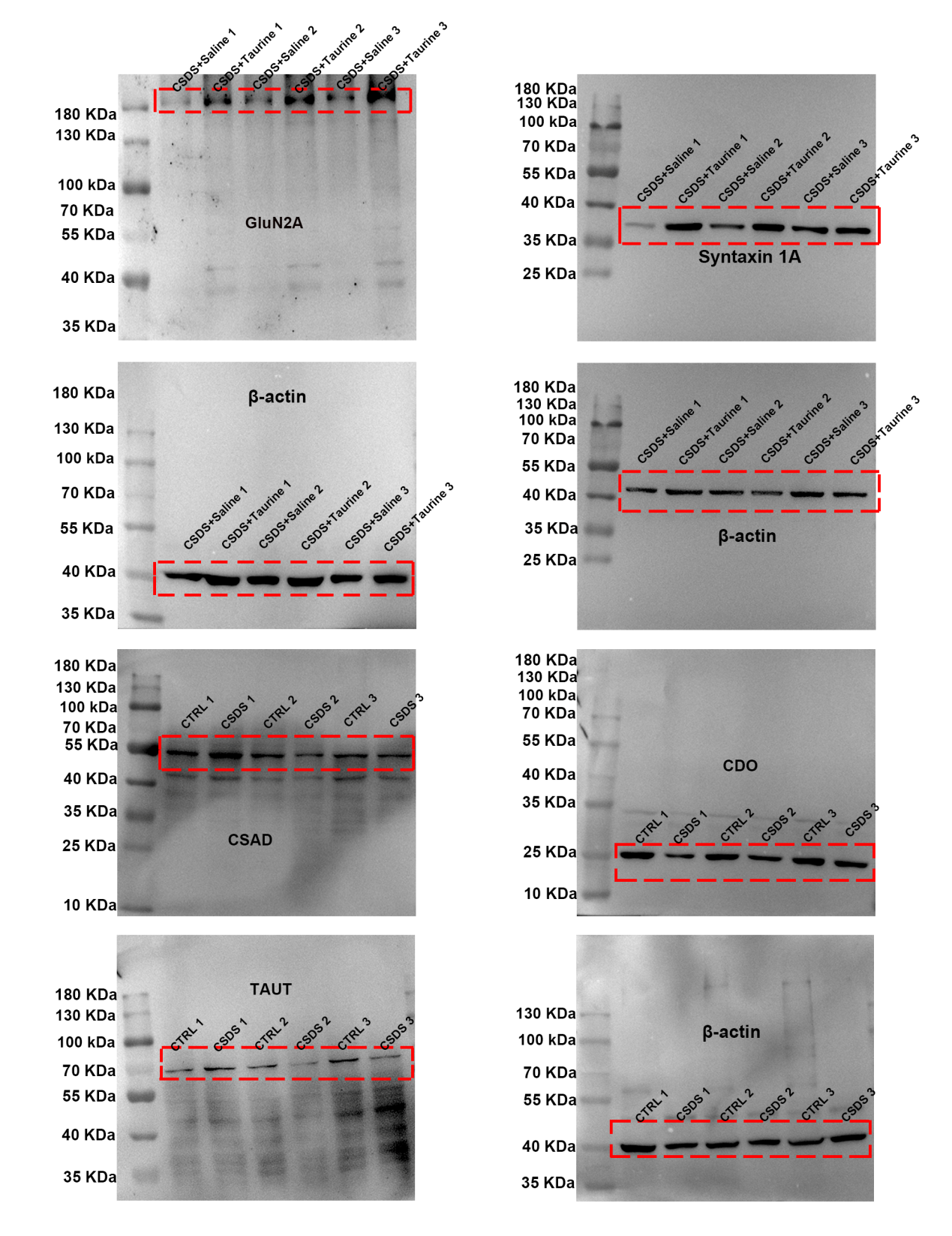


**Supplementary Fig. 4 Full gel-Western blots** Full gels displaying all proteins and the corresponding protein ladders.
